# Fast Dating Using Least-Squares Criteria and Algorithms

**DOI:** 10.1093/sysbio/syv068

**Published:** 2015-09-30

**Authors:** Thu-Hien To, Matthieu Jung, Samantha Lycett, Olivier Gascuel

**Affiliations:** ^1^Institut de Biologie Computationnelle, LIRMM, UMR 5506 CNRS – Université de Montpellier, France;; ^2^IGBMC (Institut de Génétique et de Biologie Moléculaire et Cellulaire), INSERM, U596, CNRS, UMR7104, Université de Strasbourg, Illkirch, France;; ^3^Institute of Evolutionary Biology, University of Edinburgh, Ashworth Laboratories, Edinburgh, UK

**Keywords:** Active-set method, algorithms, computer simulations, dating, influenza (H1N1), least-squares, linear algebra, molecular clock, serial data, substitution rate estimation, temporal precedence constraints, viruses

## Abstract

Phylogenies provide a useful way to understand the evolutionary history of genetic samples, and data sets with more than a thousand taxa are becoming increasingly common, notably with viruses (e.g., human immunodeficiency virus (HIV)). Dating ancestral events is one of the first, essential goals with such data. However, current sophisticated probabilistic approaches struggle to handle data sets of this size. Here, we present very fast dating algorithms, based on a Gaussian model closely related to the Langley–Fitch molecular-clock model. We show that this model is robust to uncorrelated violations of the molecular clock. Our algorithms apply to serial data, where the tips of the tree have been sampled through times. They estimate the substitution rate and the dates of all ancestral nodes. When the input tree is unrooted, they can provide an estimate for the root position, thus representing a new, practical alternative to the standard rooting methods (e.g., midpoint). Our algorithms exploit the tree (recursive) structure of the problem at hand, and the close relationships between least-squares and linear algebra. We distinguish between an unconstrained setting and the case where the temporal precedence constraint (i.e., an ancestral node must be older that its daughter nodes) is accounted for. With rooted trees, the former is solved using linear algebra in linear computing time (i.e., proportional to the number of taxa), while the resolution of the latter, constrained setting, is based on an active-set method that runs in nearly linear time. With unrooted trees the computing time becomes (nearly) quadratic (i.e., proportional to the square of the number of taxa). In all cases, very large input trees (>10,000 taxa) can easily be processed and transformed into time-scaled trees. We compare these algorithms to standard methods (root-to-tip, r8s version of Langley–Fitch method, and BEAST). Using simulated data, we show that their estimation accuracy is similar to that of the most sophisticated methods, while their computing time is much faster. We apply these algorithms on a large data set comprising 1194 strains of Influenza virus from the pdm09 H1N1 Human pandemic. Again the results show that these algorithms provide a very fast alternative with results similar to those of other computer programs. These algorithms are implemented in the LSD software (least-squares dating), which can be downloaded from http://www.atgc-montpellier.fr/LSD/, along with all our data sets and detailed results. An Online Appendix, providing additional algorithm descriptions, tables, and figures can be found in the Supplementary Material available on Dryad at http://dx.doi.org/10.5061/dryad.968t3.

## Introduction

The explosion of genetic data and progress in phylogenetic reconstruction algorithms has resulted in increasing utility and popularity of phylogenetic analyses. Data sets with thousands of taxa are becoming more and more common, especially amongst virus evolution studies. Moreover, a number of studies have used molecular-dating techniques to tackle a wide range of biological questions, for example, in systematics for timing the tree of life (Hedges and Kumar 2009; Jetz et al. 2014), in epidemiology to trace back the phylodynamics and phylogeography of epidemics (Grenfell et al. 2004; Volz et al. 2013), and in functional genomics to decipher orthology/paralogy relationships within gene families and improve reconciliation inferences (Akerborg et al. 2009; Doyon et al. 2011; Rasmussen and Kellis 2012).

Currently, the most popular dating approaches are based on sophisticated probabilistic models, most often implemented in the Bayesian framework and able to account for complex priors (Thorne and Kishino 2002; Rannala and Yang 2007; Drummond and Rambaut 2007; Guindon et al. 2010). Maximum-likelihood methods have also been designed to deal with simpler models (Rambaut 2000). Corresponding computer programs take a sequence alignment and a set of known dates as input and return a time-scaled tree, with estimates of the substitution rate(s) and of the dates of all tree nodes. Some programs (e.g., PAML, Rannala and Yang 2007) perform calculations on a fixed, user-supplied tree, while others (e.g., BEAST, Drummond and Rambaut 2007; Drummond et al. 2012) infer the tree from the sequence alignment. These programs typically contain several submodels, which describe the substitution process (e.g., GTR, Γ distribution of rates across sites, etc.), the tree (e.g., coalescent, constant or varying population size, birth–death, etc.), priors on the parameter values and, most importantly regarding dating, the molecular clock. We distinguish the strict molecular clock (SMC) model, where the substitution rate is assumed to be constant across all tree branches, and uncorrelated and correlated relaxed-clock models. With uncorrelated models, the rate associated with each branch is drawn independently from a common underlying distribution; these models are commonly used with fast-evolving species over short time periods, typically with viruses for which there is no strong evidence of rate correlation among branches (Drummond et al. 2006). With correlated (also called autocorrelated) models, the rate distribution for a particular branch depends on the rate value of the neighboring branches; the use of correlated models seems to be the preferred choice with large groups of slowly evolving species, for example mammals, where it has been demonstrated that some subgroups evolve faster than others (e.g., the rodents, Douzery et al. 2003). However, the advantages and limitations of this large variety of models is still a question of debate (Drummond et al. 2006; Lepage et al. 2007; Battistuzzi et al. 2010). All these models and methods have shown to be useful in a number of studies, but they are computationally intensive, making it virtually impossible to deal with the larger data sets available today, even when using sophisticated implementations and powerful computers (Ayres et al. 2012). Typically, days of computations are required to analyze a few hundred taxa, although faster approaches are available, using complex algorithmic approaches (Akerborg et al. 2008; Guindon et al. 2010) and multinormal approximations of the likelihood function (Thorne et al. 1998).

Here we are interested in dating very large phylogenies, typically with a thousand tips or more, a need that is becoming increasingly common, for example, in molecular epidemiology. We propose distance-based algorithms to estimate rates and dates, a mathematical and computational framework that has proven to produce fast and fairly accurate tools in phylogenetics (e.g., NJ, Saitou and Nei 1987). Several distance-based (as opposed to sequence-based, see above) dating methods have already been proposed. Most of these methods deal with time calibration points, where the dates of certain ancestral nodes in the tree are known, possibly with uncertainty (e.g., min–max values), and all of the tree tips are contemporaneous. These methods input a rooted tree with time calibration points, and return a time-scaled, ultrametric tree. PATHd8 (Britton et al. 2007) and the Tamura et al. (2012) method use smoothing and averaging techniques to accommodate for local rate variations. Xia and Yang's (2011) method assumes a SMC or two different local clocks, and achieves least-squares estimations under these assumptions. Sanderson's (1997, 2002) approach is based on a penalized-likelihood criterion to account for the autocorrelation of rates, combined with standard optimization techniques (see also TreePL, Smith and O'Meara 2012). Based on computer simulations, these fast methods were shown to be accurate by their authors, producing time-scaled trees similar to those obtained using sequence-based approaches.

The focus of the present study is on serial phylogenies, where the tips of the tree have been sampled through times. Such phylogenies are common with fast-evolving organisms (e.g., human immunodeficiency virus (HIV)), where a few years of evolution induce significant changes at the sequence level (Drummond et al. 2003a). Serial phylogenies are also used with ancient DNA (Lambert et al. 2002). Moreover, close relationships exist between the calibration-points and dated-tips approaches (Ronquist et al. 2012). Several methods have been proposed in this framework. One of the very first is root-to-tip regression (RTT) (Shankarappa et al. 1999; Drummond et al. 2003b): assuming a SMC, the root-to-tip distance in the input tree should be proportional to the corresponding elapsed time; then, a standard regression of the root-to-tip distance for every tip as a function of its date provides estimates of the substitution rate (regression slope) and root date (intercept with X-axis). This method is very fast and can be extended to unrooted trees by searching among all tree branches for the best root position, according to some numerical criterion (e.g., the sum of regression residues, to be minimized). However, this method does not provide estimates for the dates of internal nodes, and thus does not output time-scaled trees. The same holds for TREBLE (Yang et al. 2007), which is a triplet-based alternative to RTT that is also able to process unrooted trees. To obtain date estimates of the internal nodes, sUPGMA (Drummond and Rodrigo 2000) combines a regression method to estimate the substitution rate in a first step, corrects the non-contemporaneous tips into contemporaneous tips in a second step and then uses UPGMA (Sokal and Michener 1958) to compute the tree. Unlike the former approaches, Langley and Fitch's (LF; 1974) method uses an explicit model. The LF method assumes a SMC with a constant substitution rate, and models the number of substitutions along each branch of the tree by a Poisson distribution. The estimates of the global substitution rate and of the internal node dates are then obtained by maximizing the likelihood of the input, rooted tree. LF is implemented in r8s (Sanderson 2003).

In this article, we study a model analogous to LF's, but using a normal approximation that allows for a least-squares approach, and show that this model is robust to uncorrelated violations of the molecular clock. Using the tree (recursive) structure of the problem at hand, and the close relationships between least-squares and linear algebra, we propose very fast algorithms to estimate the substitution rate and the dates of all internal tree nodes. With rooted trees, the time complexity is nearly linear (i.e., proportional to the number of taxa), while with unrooted trees, it becomes nearly quadratic (i.e., proportional to the square of the number of taxa). In both cases, very large trees (>10,000 taxa) can easily be processed and transformed into time-scaled trees. The article is organized as follows: we first define the model and show its ability to handle uncorrelated rate variations among tree branches, as is commonly assumed with virus data. We then present our two main algorithms, distinguishing the unconstrained setting and the case where the temporal precedence constraints (i.e., an ancestral node must be older than its daughter nodes) are accounted for. Last, we compare these algorithms to standard approaches using simulated data and a large influenza data set. Our algorithms are implemented in the LSD program (least-squares dating), which can be downloaded (along with all data and results reported here) from http://www.atgc-montpellier.fr/LSD/ (last accessed October 2015). An Online Appendix, providing additional algorithm descriptions, tables, and figures, can be found in the in the Supplementary Material available on Dryad at http://dx.doi.org/10.5061/dryad.968t3.

## Models and Algorithms

### Preliminaries and Notation

Our algorithms take as input a binary phylogenetic tree with branch lengths, inferred by any tree building program, and sampling dates associated with the taxa. As our algorithms are very fast, it is consistent to combine them with fast tree-building methods, for example distance-based methods (e.g., NJ, Saitou and Nei 1987, or FastME, Desper and Gascuel 2002, Lefort et al. 2015), but more accurate results are expected from trees obtained using maximum-likelihood (ML) methods (e.g., PhyML, Guindon and Gascuel 2003, Guindon et al. 2010). However, we shall see that results obtained with both approaches are close. The algorithms accept a rooted or unrooted tree, and for unrooted trees we propose a method to estimate the root position, though simulations show that the use of an outgroup is generally preferable. In the following, we first assume that the tree is rooted, and then summarize the rooting procedure, which is described in more details in the Online Appendix (available as Supplementary Material on Dryad at http://dx.doi.org/10.5061/dryad.968t3).

Given a set of n serially dated sequences, let R be the input rooted binary phylogenetic tree on these sequences with known branch lengths. Enumerate the internal nodes of R by 1,2,…n−1 and the leaves by n, n+1,…,2n−1. Node 1 corresponds to the root. The date of node i is denoted by $t_{i}$. So $t_{n}$, $t_{n+1},\ldots ,t_{2n-1}$ are known. Times are measured from the origin, that is, $t_{i}⩾t_{j}$ when i is more recent than j.

For every node i different from the root (i=1), let a(i) be the parent node of i. For every internal node i, let $s_{1}(i)$ and $s_{2}(i)$ be the two direct descendants of i. Let $b_{i}$ be the length of the branch (i,a(i)); $b_{i}$ is an estimate of the number of substitutions per site that occurred along the branch from time $t_{a(i)}$ to $t_{i}$. With a SMC, the substitution rate (i.e., the expected number of substitutions per site per time unit) along the tree is constant and is denoted as ω. The goal of our algorithms is to estimate the substitution rate and the dates of all internal nodes, that is $\left(\omega ,t_{1},\ldots ,t_{n-1}\right)$.

### Probabilistic Model and Objective Function

We use a Gaussian model, which is closely related to that proposed by Langley and Fitch (1974). Assuming a SMC, the expected branch length $E(b_{i})$ is equal to ω times the time interval $\left(t_{i}-t_{a(i)}\right)$. Due to sampling noise and estimation errors, the branch length estimate $b_{i}$ (available in input tree R) can be expressed as:

(1) $$b_{i}=\omega \left(t_{i}-t_{a\left(i\right)}\right)+\epsilon_{i},$$

where $\epsilon_{i}$ is the noise (error) term. Langley and Fitch's (1974) method assumes a Poisson model for $\epsilon_{i}$, which is biologically meaningful (at least with low substitution rates and simple mutation processes). Here, we use a normal approximation for the distribution of the noise term $\epsilon_{i}$ (such an approximation is quite standard in computational statistics to accelerate the calculations, with a huge number of successful applications in many domains, and sound justifications related to the Law of Large Numbers). We thus assume:

$$\epsilon_{i}=N\left(0,\sigma_{i}^{2}\right),$$

where $N(0,\sigma_{i}^{2})$ denotes the normal distribution with mean 0 and variance $\sigma_{i}^{2}$. A limit of this model is that short branches may be negative according to Equation (1), but we impose positivity using temporal precedence constraints (see below). As evolution is independent from one branch to another, we consistently assume that the noise terms are mutually independent. The weighted least squares (WLS) criterion to be minimized (proportional to the log-likelihood assuming this model) is given by:

(2) $$\varphi \left(\omega ,t_{1},\ldots ,t_{n-1}\right)=\sum_{i=2}^{2n-1}\frac{1}{\sigma_{i}^{2}}{\left(b_{i}-\omega (t_{i}-t_{a(i)})\right)}^{2}.$$

One difficulty with such a WLS criterion lies in the variance terms $\sigma_{i}^{2}$, which are unknown and depend on the (unknown) branch lengths and possibly on some model parameters (e.g., Γ distribution of site rates). Fitch and Margoliash's (1967) tree inference method use the square of the pairwise evolutionary distance estimate. We use here another standard approach (for discussion, see Gascuel 1997) derived from the Poisson nature of the substitution process, where

(3) $$\sigma_{i}^{2}=\frac{E(b_{i})}{s}=\frac{\omega \left(t_{i}-t_{a(i)}\right)}{s}\;and\;\hat{\sigma_{i}^{2}}=\frac{b_{i}}{s},$$

with s being the sequence length.

However, the limit of such variance estimates is that overconfidence is given on very short branches, while their short length may be due to sampling randomness or estimation errors. For example, with a null branch length estimate ($b_{i}=0$), we have an infinite weight in Equation (2). This makes the method inapplicable, while the observation that $b_{i}=0$ most likely is due to the limited amount of sites available. To avoid this problem, we use the following additive smoothing for the variance estimates:

(4) $$\hat{\sigma_{i}^{2}}=\frac{b_{i}+c/s}{s},$$

where c is a constant. The higher c is, the closer we are to equal variances, that is, ordinary least squares (OLS). A value of c=1 corresponds to Laplace's Rule of Succession, which is commonly used to estimate probabilities with limited numbers of observations (with short branches, $b_{i}$ is very close to a frequency of observed differences, or p-distance, and $E(b_{i})$ to the corresponding probability). Simulation experiments (not shown) indicate that c=1 is not large enough and that c=10 provides best average results; this is the default value in our computer program, but c can be chosen by the user.

This model accommodates some violations of the molecular clock. Assume a simple model (similar to Drummond et al. 2006; see also Thorne et al. 1998) where the rate $\omega_{i}$ attached to the branch (i,a(i)) follows a normal distribution $N(\omega ,\xi^{2})$. Moreover, assume a simple model for $b_{i}$ where $\epsilon_{i}$ in Equation (1) does not depend on the branch specific rate $\omega_{i}$, but on its expectation ω, that is

$$b_{i}=\omega_{i}\left(t_{i}-t_{a\left(i\right)}\right)+N\left(0,\frac{\omega (t_{i}-t_{a(i)})}{s}\right).$$

Then, it is easily seen that

$$b_{i}=\omega \left(t_{i}-t_{a\left(i\right)}\right)+N\left(0,\xi^{2}{\left(t_{i}-t_{a\left(i\right)}\right)}^{2}+\frac{\omega (t_{i}-t_{a(i)})}{s}\right).$$

In other words, $b_{i}$ follows a normal distribution having a similar form as Equation (1), but the error term incorporates an additional factor (*i.e*., $\xi^{2}{\left(t_{i}-t_{a(i)}\right)}^{2})$, the relevance of which may be tested against the SMC. Moreover, the variance term is an increasing function of $b_{i}$, as in Equation (3), meaning that using our algorithms with uncorrelated violations of the molecular clock is still well founded.

To summarize, our model (Eq. (1)) is a normal approximation of the LF model and it naturally accommodates uncorrelated variation of rates across branches. This corresponds to the default option in several programs (e.g., BEAST), which have shown their accuracy and usefulness with numerous data sets (typically viruses, see section ‘Introduction’). We certainly do not pretend that this model depicts all the complexity of sequence evolution, but it makes possible very efficient calculations with little loss in terms of estimation accuracy, as described later.

### Outline of the Approach

The rate ω is positive, and we can fix in LSD the minimum value of the estimated rate to $\hat{\omega }⩾\omega_{min}>0$. Moreover, time is measured forward from the root to the tips of the tree, so it must satisfy the temporal precedence constraints $t_{i}⩾t_{a(i)}$ for every node i that is not the tree root (i=1). In other words, any daughter node (i>1) is more recent than its parent node (a(i)). This is an obvious requirement, analogous to the positivity of branch lengths in phylogenetic trees. However, not all dating methods comply with this requirement (e.g., see our example below with BEAST and the influenza data set), just as some phylogenetic algorithms (e.g., NJ) infer trees with negative branch lengths. The reasons for this are mostly computational. Imposing positivity constraints has a computational cost, as we shall see below in our dating context.

The estimates are obtained by minimizing the objective function φ defined in Equation (2). By using $\beta_{i}=\omega t_{i}$ for every node i=1,…,n−1 (*i.e*. i is an internal node) and $w_{i}=1/\sigma_{i}^{2}$, the function φ in Equation (2) becomes:

(2b) $$\begin{array}{c} \Psi \left(\omega ,\beta_{1},\ldots ,\beta_{n-1}\right)=\sum_{i=2}^{n-1}w_{i}{\left(b_{i}-\beta_{i}+\beta_{a(i)}\right)}^{2}\\ +\sum_{i=n}^{2n-1}w_{i}{\left(b_{i}-\omega t_{i}+\beta_{a(i)}\right)}^{2}. \end{array}$$

This function is a convex quadratic form (O'Meara 2000) and has a unique minimum (see Proof in the Online Appendix). Therefore, Equation (2) also has a unique minimum. However, to improve numerical precision our algorithms use Equation (2) and not Equation (2b), as in Equation (2b) we have to divide the variables $\beta_{i}$ by ω (another variable) to obtain the $t_{i}$, which are the true variables of interest.

We propose two different algorithms. One takes into account the temporal precedence constraints, while the other does not. For each algorithm implemented in our computer program LSD, we have two versions: weighted, where each term in Equation (2) is associated with a weight denoted $w_{i}=1/\hat{\sigma_{i}^{2}}$ (cf. Eq. (4)), and unweighted (all $w_{i}$ are equal and set to 1). We present the weighted versions in the following, as the unweighted versions are simply obtained by fixing the $w_{i}$ to 1.

### Linear Dating (LD) Algorithm, Without Constraints

Let $B'=(b_{2}^{'},\ldots ,b_{2n-1}^{'})$, where:

$$\{\begin{array}{cc} b_{i}^{'}=b_{i}, & for\;i=2,\ldots ,n-1,\\ b_{i}^{'}=\omega t_{i}-b_{i}, & for\;i=n,\ldots ,2n-1. \end{array}$$

Then, Equation (1) becomes:

$$\{\begin{array}{cc} b_{i}^{'}=\omega \left(t_{i}-t_{a\left(i\right)}\right)+\epsilon_{i}, & for\;i=2,\ldots ,n-1,\\ b_{i}^{'}=\omega t_{a(i)}+\epsilon_{i}, & for\;i=n,\ldots ,2n-1. \end{array}$$

These equations can be rewritten, using matrix notation, as B'=ωAT+E, where $T=(t_{1},\ldots ,t_{n-1})$, E is the error (noise) vector, and A is a (2n−2)×(n−1) matrix, which depends on the topology of R, such that for any i=1,…,2n−2 and j=1,…,n−1, we have:

$$A_{ij}=\{\begin{array}{cc} 1, & \;if\;(i+1<n\;and\;j=i+1)\;or\;(i+1⩾n\;and\\ & \;\;j=a(i+1)),\\ -1, & \;if\;i+1<n\;and\;j=a(i+1),\\ 0, & \;otherwise. \end{array}$$

The objective function (Eq. (2)) is then written as $\varphi ={\left(B'-\omega AT\right)}^{T}W(B'-\omega AT)$, where $W=(w_{2},\ldots ,w_{2n-1})$ is the diagonal matrix of inversed variances. By the pseudo-inverse method, the estimates of ω and T that minimize φ, satisfy $\omega (A^{T}WA)T=A^{T}WB^{\prime }$. The latter equation is equivalent to the following system of equations:

(5.1) $$\begin{array}{c} t_{1}=\frac{1}{w_{s_{1}(1)}+w_{s_{2}(1)}}[w_{s_{1}(1)}\left(t_{s_{1}(1)}-\frac{b_{s_{1}(1)}}{\omega }\right)\\ +\;w_{s_{2}(1)}\left(t_{s_{2}(1)}-\frac{b_{s_{2}(1)}}{\omega }\right)], \end{array}$$

(5.i) $$\begin{array}{c} t_{i}=\frac{1}{w_{s_{1}(i)}+w_{s_{2}(i)}+w_{i}}[w_{s_{1}(i)}\left(t_{s_{1}(i)}-\frac{b_{s_{1}(i)}}{\omega }\right)\\ +w_{s_{2}(i)}\left(t_{s_{2}(i)}-\frac{b_{s_{2}(i)}}{\omega }\right)+w_{i}\left(t_{a(i)}+\frac{b_{i}}{\omega }\right)],\\ for\;i=2,\ldots ,n-1. \end{array}$$

This system of Equations (5) can also be obtained by taking the first-order derivatives of φ with respect to each variable $t_{1},\ldots ,t_{n-1}$. Based on Equation (1), $t_{i}=t_{s_{1}(i)}-b_{s_{1}(i)}/\omega +\epsilon_{s_{1}(i)}/\omega$. Consequently, Equations (5) mean that the estimate of $t_{i}$ is equal to the weighted average of its estimates with respect to all $i^{\prime }$s neighbors (2 for tree root (i=1) in Eq. (5.1), and 3 for other internal nodes (i=2,…,n−1) in Eqs. (5.i)). The resolution of Equations (5) can be achieved in linear time (i.e., O(n), where n is the number of tree tips), while solving such a system with generic tools requires cubic time (i.e., $\text{O}(n^{3}))$. The technical details of the LD algorithm are given in the Online Appendix. The main idea is to simplify progressively this system (Eq. (5)) by recursive replacements using specific tree traversals. After the first, bottom-up set of replacements, we have

(6.i) $$\begin{array}{l} t_{i}=x_{i}t_{a(i)}+y_{i}+\frac{z_{i}}{\omega },\;\text{for}\;i=2,\ldots ,n-1,\\ \text{where}\;x_{i},y_{i},z_{i}\;\text{are}\;\text{constants}. \end{array}$$

After the second, top-down set of replacements, we obtain

(7.i) $$\begin{array}{l} t_{i}=u_{i}+\frac{v_{i}}{\omega },\;\text{for}\;i=1,\ldots ,n-1,\\ \text{where}\;u_{i}\;\text{and}\;v_{i}\;\text{are}\;\text{constants}. \end{array}$$

By using Equations (7) into Equation (2), φ becomes a quadratic function of one variable ω. Then, it is easy to compute the unique $\hat{\omega }$ value that minimizes this function. If $\hat{\omega }<\omega_{min}$, then we set $\hat{\omega }=\omega_{min}$ (optimality is shown in the Online Appendix). Last, ω in Equations (7) is replaced by $\hat{\omega }$ to obtain all date estimates $\hat{t_{i}}$.

This algorithm can be extended to non-binary trees. However, nothing guarantees that the date estimates satisfy the temporal precedence constraints. This is why we designed the QPD (quadratic programming dating) algorithm, which we describe now.

### QPD Algorithm

QPD is based on an active-set method, which is commonly used to solve optimization problems with linear constraints (Nocedal and Wright 2006). Let $x=(\omega ,t_{1},\ldots ,t_{n-1}$); the function to minimize is φ(x) defined by Equation (2), subject to the constraints $t_{i}-t_{a(i)}⩾0$, for i=2,…,2n−1. For the sake of simplicity, we do not include the ($\omega ⩾\omega_{min}>0$) constraint, as it is already accounted for in the LD algorithm, which is part of QPD. x is a “feasible” point if and only if it satisfies all the constraints. A constraint i is “active” at x if and only if $t_{i}=t_{a(i)}$. The active-set method applied to our problem can be summarized as follows (see the Online Appendix for details): starting from a feasible point x with C being the set of active constraints, we compute the minimal solution of Equation (2) with respect to C, that is, the minimal solution such that $t_{i}=t_{a(i)}$, for every i∈C. We thus have to calculate the stationary point $\left(x^{*},\lambda^{*}\right)$ of the Lagrange function:

(8) $$\Gamma \left(x,\lambda \right)=\varphi (x)-\sum_{i\in C}\lambda_{i}\left(t_{i}-t_{a\left(i\right)}\right).$$

We then check if: (i) some constraints are violated in $x^{*}$, and (ii) all constraints in C are useful. C is updated accordingly, by relaxing the “most useless” constraint and adding the “most violated” one. The algorithm stops when all constraints in C are useful and no more constraints are violated (Karush–Kuhn–Tucker (KKT) conditions, Boyd and Vandenberghe 2004). With strictly convex quadratic functions, this method is ensured to converge to the unique global minimum (Nocedal and Wright 2006). Although Equation (2) does not comply with these requirements, a proof of QPD convergence to the unique minimum is provided in the Online Appendix.

The active-set method is especially efficient here, because we can find the stationary point of the Lagrange function (Eq. (8)) in linear time. Indeed, $x^{*}$ is computed by a modified version of the LD algorithm, which applies to a new tree obtained from the input tree R by collapsing the branches corresponding to the active set C. Then, $\lambda^{*}$ can also be calculated in linear time (Online Appendix).

The time complexity of QPD is O(f×n), where f is the number of iterations needed to reach the optimal solution, and n is the number of taxa. f depends on the data and the chosen starting point. We use here the LD algorithm, initializing C with the violated constraints $\left(t_{i}<t_{a(i)}\right)$ in the LD solution, which are combined to obtain a feasible point. In our experiments (described below), QPD performs 3 iterations on average with simulated trees of 110 taxa, and 69 iterations with an H1N1 influenza data set of 891 taxa. Although, it is difficult to extrapolate from these experiments, it seems that in practice f is much smaller than n, and thus the computing time of QPD appears to be nearly linear.

### Estimating the Root Position for Unrooted Trees

Given an unrooted tree, we estimate the root position by searching for the point in the tree that minimizes the objective function (Eq. (2)) when the tree is rooted at this point. A similar approach is used in RTT-based Path-O-Gen software (Rambaut 2007). In essence, this is the point that makes the tree the most molecular clock-like. Let R be an unrooted tree with the internal nodes enumerated from 2 to n−1, and the external nodes from n to 2n−1. Let r be a point on a branch $\left[r_{1},\;r_{2}\right]$ of length b. Let $t_{r}$ be the date of r assuming r is the tree root, and μ a variable in [0,1] such that the length of branch $\left(r_{1},\;r\right)$ is equal to μb; then, the objective function (Eq. (2)) of the tree rooted at r becomes:

(9) $$\begin{array}{c} \;\phi \left(t_{r},t_{2}\ldots ,t_{n-1},\omega ,\mu \right)\\ ={\left(\mu b-\omega (t_{r_{1}}-t_{r})\right)}^{2}+{\left(\left(1-\mu )b-\omega (t_{r_{2}}-t_{r}\right)\right)}^{2}\\ +\sum_{i\neq r_{1},r_{2}}{\left(b_{i}-\omega (t_{i}-t_{a(i)})\right)}^{2}. \end{array}$$

Note that we do not use weights (variances) in the objective function, since weights depend on their associated branch lengths, which are unknown for the two branches containing the assumed root. Optimizing this function without and with constraints can be done by slightly modifying the LD and QPD algorithms, without changing their time complexities. The technical details are given in the Online Appendix. For each branch, we calculate the root position (μ) which minimizes Equation (9), and then take the minimum point among all branches. Therefore, the time complexity is n times that of the LD and QPD algorithms. Since LD is linear, the corresponding rooting algorithm is quadratic. For QPD, to avoid exploring all branches, which could be time consuming with large trees, we pre-estimate the position of the root using LD, and then we use QPD to perform a greedy search for the local minimum around that position. This rooting method is also applicable when all tips are contemporaneous, thus representing a new alternative to the standard rooting methods (midpoint, minimum-variance, etc.).

## Results with Simulated Data

### Data Simulation

We implemented a tree generator based on a simple birth–death model with periodic sampling times, mimicking typical intrahost studies with yearly sampling, or (interhost) epidemic surveillance through time. We first assumed a SMC, and then a lognormal relaxed molecular clock (RMC). Let us start with SMC. At time t=0, there is one single individual (n=1), which is iteratively subdivided. At each step, one of the n individuals is randomly selected and divided into two individuals, resulting in n+1 individuals. The elapsed time between the previous division event and the new one is equal to 1/n (i.e., like the standard Yule tree, where the expected time is equal to 1/n). This process is continued until we have 1000 individuals. Then we proceed with sampling and death: the evolution of a number of individuals (e.g., 750) is stopped, most of them (e.g., 725) are removed from the tree (or “culled”), while the remaining ones (e.g., 25) are retained to be the sampled individuals of the first sampling time. The process continues with the nonculled and nonsampled individuals (250 in our example), which are further divided using the same Yule-type rule until we again have 1000 individuals to be sampled, culled, or conserved for the next step. The whole process is continued until we attain the desired number of sampling times. The final set of sampled individuals is exactly the taxon set (or leaves) of the final tree. This tree is then rescaled so that the time between the first and the last sampling time is 20 years, with the root date being zero. An advantage of this scheme is that the time elapsed from one sampling time to the next one is constant, thus emulating the sampling of DNA sequences from an evolving population on a regular basis, as opposed to standard birth–death tree generators (Stadler 2010). Moreover, with birth–death trees the divergence times vary among replicates, while here we use fixed divergence times for easy estimation of method accuracy and presentation of the results.

**Figure 1.**
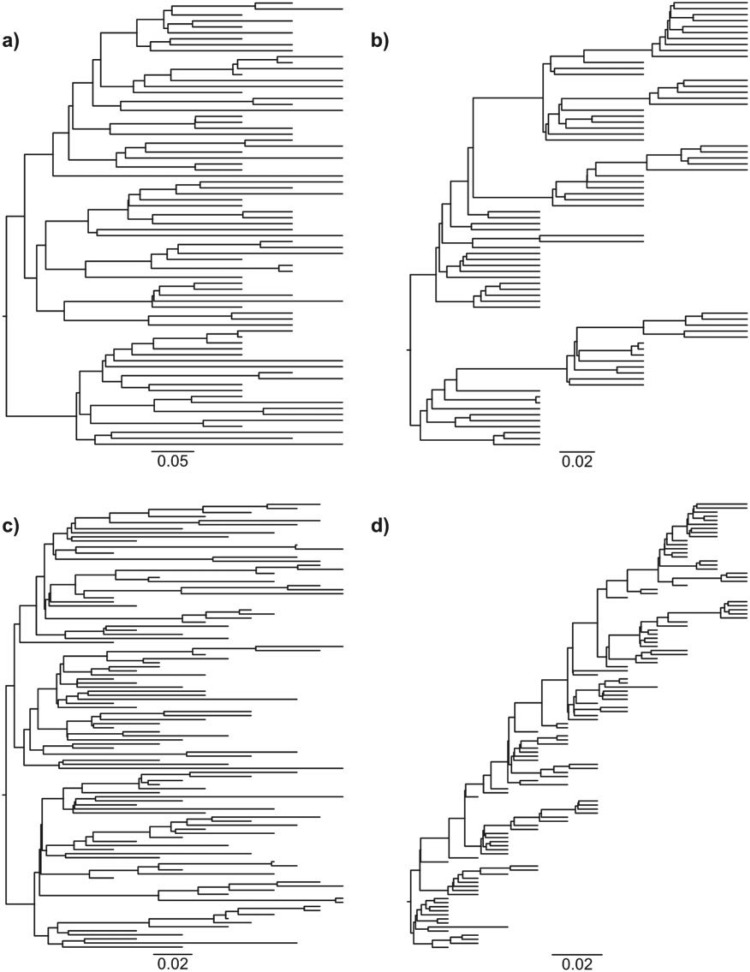
Examples of simulated trees. Four examples of trees extracted from our simulated data sets. Trees a) and c) are intended to simulate inter-host evolution of HIV (one tip per host; 750/1000 of the strains are removed at each sampling date). Trees b) and d) are intended to simulate intra-host evolution of HIV, with its typical “ladder shape” (all tips from a single host; 995/1000 of the strains are removed at each sampling date). Trees a) and b) have each 3 sampling dates with 25 sampled strains each. Trees c) and d) have each 11 sampling dates with 10 sampled strains each. See text and Volz et al. (2013) for explanations.

We generated two kinds of trees, intended to simulate interhost and intrahost HIV evolution (Volz et al. 2013), by using two death rates (ratio of individuals removed at each sampling time): 750/1000 was used for interhost trees, and 995/1000 for intrahost (typically ladderized) trees. For each, we used 3 sampling times (separated by 10 years) with 25 selected individuals at each time, and 11 sampling times (separated by 2 years) with 10 selected individuals at each time. See Figure 1 for examples of trees. Additionally, we added one outgroup to simulate the search for the root position using the standard outgroup-based approach. The length of the branch from the ingroup root to the outgroup was three times the length from the ingroup root to the nearest ingroup leaf. Last, to simulate sequence evolution, we used the substitution rate ω to obtain the length of each branch $b_{i}=\omega (t_{i}-t_{a(i)}$), corresponding to the expected number of substitutions per site along that branch; ω was equal to 0.006 substitutions per site and per year, which is similar to the substitution rate of the HIV *env* gene (Bello et al. 2008). With each combination of these parameters, 100 trees were randomly generated. Hence, there are in total 4×100 SMC trees, denoted as (death rate/sampling scheme): 750/3×25, 750/11×10, 995/3×25, and 995/11×10.

To simulate trees with RMC, we used the uncorrelated lognormal model, which is one of the most widely used in BEAST (Drummond et al. 2006). For this purpose, we reused the previous trees, but multiplied every branch length by a random variable following a lognormal distribution with mean 1 and standard deviation 0.4. This value is between the estimates we obtained for *pol* and *env* HIV genes (unpublished results). We thus obtained 4×100 RMC trees.

DNA sequences of length 1000 were evolved along these trees using Seq-Gen (Rambaut and Grassly 1997), version 1.3.2x. We used the F84 model with a Γ distribution with shape parameter 1.0 and 8 rate categories, a transition/transversion rate ratio of 2.5, and nucleotide frequencies of (A, C, G, T) = (0.35, 0.20, 0.20, 0.25). These parameter values are similar to estimates already observed with the *env* region of HIV (Posada and Crandall 2001).

To assess the accuracy of the distance-based dating methods, we inferred trees from these alignments. First, we used the correct tree topology but re-estimated the branch lengths using PhyML+F84+Γ8; the aim was to measure the impact of topological errors that are unavoidable in real studies; moreover, we used these trees to assess the performance of the various methods to estimate all tree node dates, instead of the root date only. Second, we used DNADIST+F84+Γ (PHYLIP, Felsenstein 1989, v3.69) to estimate pairwise evolutionary distance matrices, and then FastME with SPR option to estimate trees (negative branch lengths were set to zero); the distance estimation parameters in DNADIST were the same as those used to generate the data with Seq-Gen; the aim was to check the accuracy of a fast distance approach, being easily bootstrapped or able to tackle very large data sets. Last, we used PhyML+F84+Γ8 with SPR option to infer both the tree topology and branch lengths, that is, a standard ML method with high accuracy, but slower than DNADIST+FastME. All these trees were used in two ways: (i) the outgroup was used to produce rooted trees, from which the outgroup was deleted; (ii) we simply removed the outgroup to obtain unrooted trees. All of our data sets (model trees, alignments, distance matrices, inferred trees, etc.) are available at http://www.atgc-montpellier.fr/LSD/.

### Methods for Comparison

These simulated data were used to assess the performance of our two methods (LD and QPD) and of three other methods: RTT, Langley-Fitch (LF*), and BEAST (BSMC with a SMC model and BRMC with a relaxed clock):

- For LD and QPD, if the tree is rooted, the program uses the given tree; the methods are then denoted as LD* and QPD*, and we use the “variance” option (WLS). Otherwise, the root position is estimated and the methods are simply denoted as LD and QPD.
- For RTT, we re-implemented the linear regression method, which takes both rooted and unrooted trees as input. Given unrooted trees, it estimates the position of the root by minimizing the sum of squared residues. Given rooted trees, the method is a standard regression and is denoted as RTT*. For dozens of data sets, we checked that our implementation gives the same result as Path-O-Gen v1.3 (Rambaut 2007). Unlike other methods used here, RTT does not estimate the dates of internal nodes but only the root date and the substitution rate.
- For LF*, we used the program implemented in the *r8s* package v1.8 (Sanderson 2003); the likelihood function was optimized thanks to Powel's algorithm (TN algorithm was much faster, but returned inconsistent results with ∼20% of our data sets); this program has no ability to search for the root position and takes only rooted trees as input, hence the notation LF*.
- For BSMC and BRMC, BEAST version 1.7 was used with HKY+Γ8 (closely related to F84+Γ8 used to simulate the data) and coalescent with constant population size tree prior. We used a SMC with an uninformative prior (*clock rate* had a uniform distribution between 0 and 1). The length of the MCMC chain was $5\times 10^{\text{6}}$ generations, with a burn-in of 10% and a sampling every $5\times 10^{\text{3}}$ generation. For the relaxed-clock data, we also used a lognormal relaxed-clock model (i.e., the model used to generate the data); the prior of the *ucld.mean* parameter had a uniform distribution between 0 and 1, and the prior of *ucld.stdev* had an exponential distribution with parameter 1/3 (default value). The MCMC chain length was increased to $20\times 10^{\text{6}}$ generations, with a burn-in of 10% and a sampling every $20\times 10^{\text{3}}$ generations. These parameter values are standard and default options were used in all of our analyses. We increased the burn-in up to 25%, but did not observe significant changes. Additional runs with several alternative priors were also performed (uniform prior in a much more narrow interval [0, 0.05] for *clock.rate* and *ucld.mean* parameters; uniform prior on the inverse of these parameters; birth–death tree prior), but without improvement, and the same held with alternative program options (Drummond A., Yanez R., personal communication). Moreover, other runs of BEAST were carried out to assess the accuracy of internal node date estimations. We then used the true rooted tree topology (otherwise date comparisons are meaningless), and forced it to be constant in BEAST, so that only the branch lengths were re-estimated, just as with PhyML (see above). The length of the MCMC chain was set to $10\times 10^{\text{6}}$ generations, with a burn-in of 10% and a sampling every $10\times 10^{\text{3}}$ generations. In all of our analyses, we used *meanRate* estimator for rate estimations with BRMC, since it was more accurate than *ucld.mean*, and *clock.rate* with BSMC; *treeModel.rootHeight* was used to estimate the root date with both BSMC and BRMC. BEAST xml and log files with the 800 simulated data sets are available at http://www.atgc-montpellier.fr/LSD/.

### Comparison Criteria

With simulated data, the true value of the parameters (substitution rate, root and node dates) are known. We used standard quadratic error measures to compare the true and estimated values and assess the accuracy of the methods being compared. An advantage of these measures is that they can be decomposed into variance and bias terms, thus indicating whether the estimation method shows some tendency to over- or underestimate the true parameter value, and whether the main source of errors is, or is not, the variance of the estimates.

- For the substitution rate, let ω be the true value, ${\hat{\omega }}_{i}$ the value estimated by a given method with the $i^{th}$ data set among m (= 100 in our experiments), and $\bar{\omega }$ the average of the m estimates. The accuracy of that method in estimating the substitution rate is measured by the relative error:
  $$\frac{1}{\omega }\sqrt{\frac{1}{m}\sum_{i=1}^{m}{\left(\omega -{\hat{\omega }}_{i}\right)}^{2}},$$
  and the relative bias is defined by:
  $$\frac{1}{\omega }\left(\bar{\omega }-\omega \right).$$
- Similar measures are used for the root date, with relative error defined by:
  $$\frac{1}{t_{c}}\sqrt{\frac{1}{m}\sum_{i=1}^{m}{\hat{t}}_{i}^{2}},$$
  and relative bias:
  $$\frac{\bar{t}}{t_{c}},$$
  where ${\hat{t}}_{i}$ is the estimated root date with the $i^{th}$ data set, $\bar{t}$ is the average root date estimate, and $t_{c}$ is the contemporary time (which is the same for all trees within each tree model); moreover, remember that the true root date is zero. These relative error terms can be interpreted as percentages; for example, a bias of −0.1 means that the true value is underestimated by 10%, in average. A basic result in estimation theory is that the square of the bias plus the variance of the estimates is equal to the mean square error. It follows that our relative bias is less than the relative error and that their difference corresponds to the relative, standard deviation of the estimates. We calculated the confidence intervals of these error measures using the bootstrap method; for each data set of 100 trees, we re-sampled 10,000 times with replacement the set of the 100 estimated values and computed the corresponding error; then, the 2.5% and 97.5% quantiles were picked up to form 95% confidence intervals.
- For the dates of internal nodes, we used the absolute error (measured in years and thus easily interpreted) defined by:
  $$\sqrt{\frac{1}{m(n-1)}\sum_{i=1}^{m}\sum_{k=1}^{n-1}{\left({\hat{t}}_{ik}-t_{ik}\right)}^{2}},$$
  where i=1,…,m represents one of the m trees, and k=1,…,n−1 is one of the internal nodes (including the root, where k=1), $t_{ik}$ is the date of the node k in the tree i and ${\hat{t}}_{ik}$ is its estimated value. Again we used the bootstrap to build confidence intervals.

### Results

The detailed results of all tested methods using above criteria are available from our web site http://www.atgc-montpellier.fr/LSD/ and in the Online Appendix.

Distance-based dating methods have negligible computing times with these data (∼0.1 seconds or less, even with unrooted trees where the root position has to be searched among all edges), except LF*, which is still fast but requires a few seconds with rooted trees. In contrast, BEAST requires a few hours with a SMC, and a dozen hours with a relaxed clock. For a fair comparison, we also have to account for tree building, as BEAST infers both the tree and the dates. However, PhyML is much faster, requiring 8 min for the largest 110-taxon trees. The computing time difference between distance-based approaches and BEAST is thus very large (see Online Appendix Supplementary Table S1 for details), but does not correspond to gains in estimation accuracy, as discussed below.

**Figure 2.**
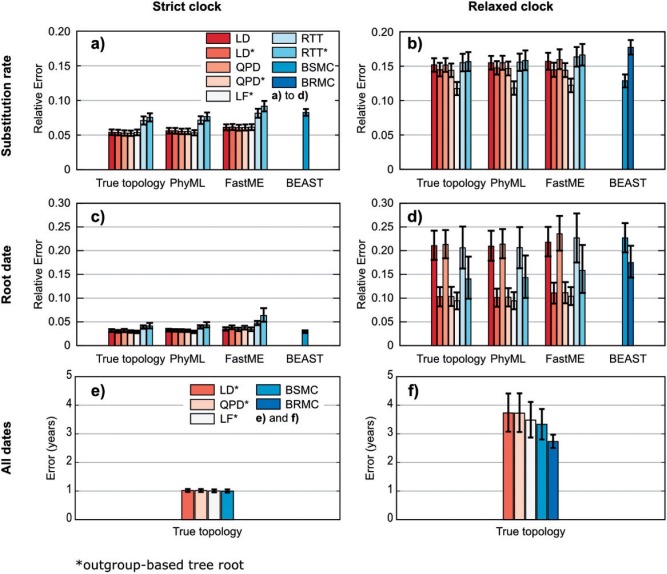
Summary results with simulated data. Panels a), c) and e) contain summary results of the trees with a SMC, panels b), d) and f) those with a lognormal, RMC. Panels a) and b) show the relative error of the substitution rate estimates, panels c) and d) show the relative error of the root date estimates, panels e) and f) show the average error (in years) of the data estimates of all tree nodes. See text for the definitions of these measures. From left to right (see legends) tested methods are: linear dating with tree root estimation (LD); linear dating with outgroup-based tree rooting (LD*); quadratic programing dating with tree root estimation (QPD); quadratic programing dating with outgroup-based tree rooting (QPD*); Langley-Fitch that uses rooted trees only (LF*); root-to-type regression with tree root estimation (RTT); root-to-type regression with outgroup-based tree rooting (RTT*); BEAST with strict molecular clock (BSMC); BEAST with lognormal, relaxed molecular clock (BRMC).

With SMC data (Fig. 2a,c,e), the relative errors are low (∼ 5%) and most methods have similar, high accuracy. RTT and RTT* are a bit less accurate than the others for both root date and rate estimations, most likely due to their overly simple model. BEAST is also behind the others regarding rate estimation, with a substantial positive bias (up to ∼10% with 995/11x10 trees, Online Appendix Supplementary Table S2), but performs well with date estimation, both for the root (Fig. 2c) and all internal nodes (Fig. 2e). As a general tendency (Fig. 2a,c, e.g., LD vs. LD*, and QPD vs. QPD*), molecular clock-based rooting produces similar results to outgroup-based rooting for both the root date and the rate, as expected since trees were generated with SMC. Surprisingly, the accuracy of rate and root date estimations are not significantly affected by topological errors: although the FastME and PhyML trees contain a substantial amount of erroneous branches, we see very little difference in accuracy between the results obtained with the true and inferred topologies. Moreover, there is almost no difference between the results obtained with FastME (topological error ∼15%, Online Appendix Supplementary Table S4) and PhyML (topological error ∼10%). This suggests the use of (much faster) FastME rather than PhyML, when the aim is not to obtain a fully correct tree topology but to quickly estimate rates and dates, or to perform bootstrap analyses. The topological accuracy of BEAST and PhyML are quite similar (Supplementary Table S4), with BEAST providing a slight advantage, meaning that the high error of BEAST in rate estimation is not due to topological errors, but to the positive bias already indicated above. BEAST results with the fixed, true topology confirm this finding (Online Appendix Supplementary Fig. S5).

With RMC data (Fig. 2b,d,f), the relative errors of all methods are much higher (from ∼10% to ∼20%) than with SMC data (∼5%). Again, the topological errors have little impact on the accuracy of rate and date estimations, and cannot explain the differences among the various methods, especially with BEAST the topological accuracy of which is still slightly better than PhyML's (Supplementary Table S4). Again, FastME and PhyML trees produce rate and date estimates showing similar accuracy. As expected the main factor is root positioning, which has a high impact on root date estimations. If the root is misplaced, the tree cannot be dated precisely. Among the methods directly inferring the root position (i.e., without outgroup), LD, QPD, and RTT show similar accuracy (poor regarding root date), while BEAST results differ depending on the clock model. With BSMC the rate is well estimated but the date is not any better than with direct distance-based approaches; with BRMC the rate is poorly estimated due to a high positive bias (>10%), but the root date is fairly well estimated. Results with the fixed, true topology confirm these findings: BEAST rate estimations are not improved (Supplementary Fig. S5), but BEAST with the RMC model is the most accurate method to estimate internal node dates (Fig. 2f), which is to be expected since the data were generated using the very same model. Moreover, the global average results (Fig. 2) hide that BEAST does well with model trees with low death rate (750/1000, Online Appendix Supplementary Figs. S1–S2), but not so with high death rate (995/1000, Online Appendix Supplementary Figs. S3–S4). Among the methods using outgroup-based rooted trees: LF* is best to estimate the rate and slightly (but not significantly) better than LD* and QPD* in estimating root and internal node dates; RTT* is worse both to estimate the rate and the root date.

Up until now, we mostly discussed average results over the four types of model trees (Fig. 2). As expected, the accuracy of the various methods differs depending on the model tree (Online Appendix Supplementary Figs. S1–S4). The accuracy of the estimates is better with the larger sample of 110 dated sequences, than with 75 sequences, and the impact is especially sensible with date estimations since we have 11 sampling times (every 2 years) instead of 3 (every 10 years). Moreover, the ladder-shaped trees (995/1000 death rate) are easier than the more star-like trees (750/1000), an outcome already observed with real data, for example from human seasonal influenza (ladder-shaped) versus HIV (star-like) epidemics (Grenfell et al. 2004). However, the global properties and the ranking of the various methods remain similar compared to average analysis (except with BEAST, see above).

Most results in these simulations were expected. Among distance-based methods, LF* has the most realistic (Poisson) model and obtains the best results; LD* and QPD* use a simplified (normal) version of the LF* model, and their results are not as good as those of LF*, although the difference is not significant in most cases; RTT* is the worst distance method, as expected since its model is too simple and does not account for the fact that the root-to-tip paths are highly correlated (Drummond et al. 2003b). The main surprise comes from the results of BEAST, expected to be the best due to its sophisticated model, being identical or very close to the data model, but in fact the results on the data sets used here do not suggest this. However, results in Figure 2 have to be interpreted with care: first, BEAST in our experiments does not use an outgroup as the best distance approaches do, and thus should be compared to the direct methods (LD, QPD, and RTT); second, BEAST shows a substantial bias with rate estimation that remains to be explained, but performs well (Fig. 2d) to very well (Fig. 2f) with dating.

Let us conclude these simulations with practical guidelines. Tree rooting is a difficult task; thus, if possible, use an outgroup and compare the results with the direct ones, obtained by assuming some (relaxed) clock model. When having a well-supported and consistent root position, use LF* or QPD*, selecting the latter with large data sets and bootstrap studies. ML trees are preferable to minimize topological errors, but (fast) distance-based trees provide nearly identical rate and date estimates. LD and QPD (resp. LD* and QPD*) have nearly identical accuracy in these simulations. However, LD and LD* violate a substantial number of temporal constraints (∼4% by more than 1 month with 110-taxon trees and RMC), and the advantage of QPD and QPD* will become even more apparent with real (imperfect) influenza data.

## Application to an Influenza Data Set

To illustrate the results of our algorithms on large data sets, we used a set comprising 1194 strains of influenza A virus subtype H1N1pdm09, which caused the first human influenza pandemic of the 21st century. The first two cases were reported in children from southern California on 21 April 2009. Soon after, other cases were reported, and by 11 June 2009, 27,000 cases of infection had been observed from 74 countries, including 141 deaths. On that date, the World Health Organization (WHO) declared a pandemic, and the end of the pandemic was declared in August 2010 (for details, see Christman et al. 2011).

Molecular epidemiology studies on this virus were performed at an early stage of the epidemic, using 242 strains collected between 30 March and 12 July 2009 (Lemey et al. 2009; Rambaut and Holmes 2009). These studies indicated that this virus has a high evolutionary rate of $4.96\times 10^{\text{-3}}$ [$4.10\times 10^{\text{-3}}$; $5.87\times 10^{\text{-3}}$] substitutions per site and per year (for concatenated hemagglutinin (HA) and neuraminidase (NA) genes), and the estimated date for the tMRCA was 27 January 2009 [29 December 2008; 22 February 2009]. This MRCA date was confirmed by Hedge et al. (2013) using 328 whole virus genomes sampled in North America before April 2010. To our knowledge, no other molecular dating study has been published on a more comprehensive set of strains sampled over a longer time period.

The (1194) strains used here were collected worldwide between 13 March 2009 and 9 June 2011 (see Online Appendix Supplementary Table S5 for further details). The A/Swine/Hong Kong/1110/2006 (subtype H1N2) strain was used as outgroup to root the phylogenetic tree. The HA gene sequences were aligned by codon using MUSCLE in MEGA 5.0 and checked manually, resulting in an alignment of 1194 (+1 outgroup) sequences and 1701 sites. As many sequences were identical but collected at different time points, we retained for each set of identical sequences only one exemplar with a sampling date equal to the average of the dates of the corresponding strains. We thus obtained 891 (+1 outgroup) different sequences, each with a unique sampling date, from which a phylogenetic tree was computed. Note that grouping identical sequences does not impact phylogeny inference (identical sequences are separated by branches of length zero) but accelerates the computations and is consistent with our dating model which has difficulty in dealing with branches of length zero but different dates at both extremities (see Eqs. (1)–(4), and notably the variance term). However, this simplification was not used with BEAST, which handles such data due to its coalescent, population genetics model.

To run our dating algorithms, we first have to infer a phylogenetic tree. Two methods were used, as in our simulation study, with different speed/accuracy tradeoff: (i) a fast distance-based method, namely FastME with the SPR option and distances estimated by DNADIST under F84+Γ (the Γ parameter was set to 1.0, as in other experiments); (ii) a more accurate but slower ML method, namely PhyML with SPR option and GTR+I+Γ4. For both methods we analyzed the ingroup sequences only and considered both the outgroup-based rooted tree, and the unrooted tree obtained by root removal. To compute confidence intervals we used the bootstrap method with 100 replicates generated with SEQBOOT from the PHYLIP package. To improve computational efficiency, the tree topology was kept constant and equal to the topology inferred using the original data set; only the branch lengths were re-estimated from the bootstrap samples.

We compared the same methods as in the previous sections, using the same options. LD and QPD were run with the “variance” (WLS) option both in the rooted and unrooted settings; they are denoted as LD* and QPD* with rooted trees. Langley-Fitch (LF*) from r8s was run with the rooted tree only, as it has no means to infer the tree root. RTT (our implementation, equivalent to Path-O-Gen v1.3, Rambaut 2007) was performed with both the unrooted (RTT) and the rooted (RTT*) trees. BEAST was run from the complete, ingroup alignment using GTR+I+Γ4, a coalescent constant population size tree prior and two molecular clock models: strict (SMC) and relaxed lognormal (RMC), with normal clock priors ($\text{mean}=4\times 10^{\text{-3}}$ substitutions per site per year (s/s/y), standard $\text{deviation}=2\times 10^{\text{-3}}$ s/s/y). Two independent MCMC chains were used per model, with a minimum of 250 million generations each, sampling every 10,000 generations. The first 25 million generations in each run were discarded as burn-in, and the Highest Posterior Density statistics for each parameter were calculated over a posterior sample of 1000 states using Tracer 1.5. Moreover, as we observed a strong discrepancy between BEAST and the other methods regarding substitution rate estimations (see below), we also launched BEAST with the cleaned data set where identical sequences were grouped (891 taxa), and using the PhyML rooted tree topology which was kept constant all along the computations, solely sampling the branch lengths and model parameters. Such use of BEAST seems to be rather uncommon, but corresponds to the way a number of other dating programs proceed, for example, PAML (Rannala and Yang 2007).

All methods except BEAST were run on our server (Intel(R) Xeon(R) X5650 @ 2.67GHz, single core, no parallelization), while BEAST was run on a Dell Precision T7500 workstation (Intel(R) Xeon(R) X5687 @ 3.6GHz CPUs, one core per model) using the Beagle library with the SSE, Double Precision, and Dynamic Rescaling options (Ayres et al. 2012). The computing times (Table 1) to obtain point estimates for the substitution rate and all node dates with distance-based approaches are very fast: at most 1 second with rooted trees for LD*, QPD*, and RTT*, and ∼1 min for LF* which is the slowest distance-based method; with unrooted trees, the methods inferring the tree root (LD, QPD, and RTT) are inevitably slower as they have to search all tree branches, but are still fast requiring less than 1 min. To obtain bootstrap intervals the computing times are multiplied by 100 as we have 100 replicates, varying from a few seconds (RTT* and LD*) to ∼1 h (LF*), with QPD* requiring ∼2 min. This shows the advantage brought by our algorithms, since both LF* and QPD* use closely related models and show similar accuracy (Fig. 2). However, the time to build trees has to be accounted for, especially when bootstraps are used. DNADIST+FastME is remarkably fast, requiring ∼1 h to infer the original and 100 bootstrap trees, while PhyML is much slower, requiring ∼4 d for the same task. To get a good posterior sample of time resolved Bayesian phylogenetic trees with the 1194 sequences requires running BEAST for a minimum of 20 d, using at least 250 million MCMC generations at approximately 2 h per million generations. For the 891 cleaned sequence set, using a fixed rooted, PhyML topology in BEAST, only 50 million MCMC generations are needed, taking 5 d at approximately 2.5 h per million generations.

**Table 1.** Computing time for the H1N1pdm09 Flu data set

	Original sample	100 bootstrap samples
*Phylogeny inference*		
Distance-based (DNADIST+FastME)	127	3434
ML (PhyML)	∼60 h	∼35 h
*Dates and rate estimation*		
LD	31	2791
LD*	<1	15
QPD	38	3329
QPD*	1	120
Root-to-tip	12	2765
Root-to-tip*	<1	2
Langley–Fitch*	54	4177
BEAST (BSMC and BRMC)	∼20 d	–
BEAST* (BSMC* and BRMC*)	∼5 d	–

Note: Time is expressed in seconds, except otherwise specified. With bootstrap samples, only the branch lengths were reoptimized; the tree topology was kept constant and equal to the topology inferred using the original alignment. BEAST was run to infer all model parameters, including the tree topology and tree root, while with BEAST* we used the PhyML rooted tree topology which was kept constant along the computations. The asterrisk (*) denotes methods using out-group based rooted trees.

**Figure 3.**
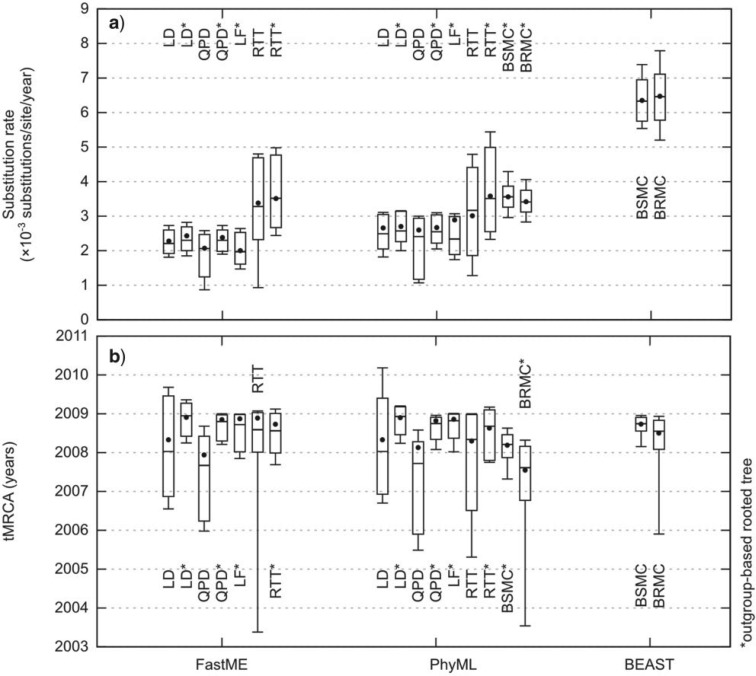
Estimations of the substitution rate (panel (a)) and tMRCA (panel (b)) with the H1N1pdm09 Flu data set. Distance-based estimation methods are run with both DNADIST+FastME and PhyML trees. LD, QPD, and RTT (root-to-tip) are run from unrooted trees and search for the best tree root position. LD*, QPD*, LF*, and RTT* are run using outgroup-based rooted trees. BEAST is run from the ingroup sequence alignment, with both a strict molecular clock (BSMC) and a lognormal relaxed clock (BRMC); BSMC and BRMC infer the tree topology and root position, while BSMC* and BRMC* use the fixed, rooted tree topology inferred by PhyML. The box plots represent the median, maximum, minimum, 97.5% and 2.5% quantiles of the bootstrap estimates with distance-based methods, and of the posterior distribution with BEAST. The distance-based point estimates and BEAST posterior means are represented by a dot.

We see little difference (Fig. 3) between the results obtained with FastME and PhyML trees, especially for the tMRCA where point and interval estimates are nearly the same for every distance-based estimation method (except root-to-tip). This strongly suggests using FastME when the focus is on rates and dates, at least for large data sets, as it is several orders of magnitude faster than PhyML. Moreover, both tree building and dating are then consistently based on similar distance-based approaches.

Regarding rate estimation (Fig. 3a), all distance-based methods provide similar results, except root-to-tip regression with faster rate estimates and much larger confidence intervals. QPD also shows relatively large intervals, likely due to the fact that it has to infer the tree root and is thus subject to more variability and possible rooting errors. However, the main fact here is that distance-based and BEAST rate estimates (obtained from the complete data set, while optimizing the tree topology) widely differ ($\sim 3.0\times 10^{-3}$ and $\sim 6.5\times 10^{-3}$, respectively, with non-overlapping confidence and credibility intervals). With simulated data we found that BEAST with the specified priors and options may overestimate the substitution rate (Online Appendix Supplementary Tables S2 and S3). We also observed similar discrepancies between both approaches on other biological data sets (results not shown). However, the gap here was so large that we ran BEAST with the cleaned data set and the fixed PhyML rooted tree topology that was used with other approaches. Then, BEAST rate estimates (BSMC* and BRMC* in Fig. 3a) became much closer to the others, being still somewhat faster ($\sim 3.5\times 10^{-3}$ instead of $\sim 3.0\times 10^{-3}$) but with mostly overlapping intervals. BEAST (combined with TREEANNOTATOR) infers a tree where ∼2% of the temporal precedence constraints are violated with the complete data set, while with the cleaned data set and the fixed PhyML rooted tree, all constraints are satisfied. The reasons for these findings are still unclear. One explanation could be that with such a large data set (>1000 sequences) BEAST has difficulty in converging on a reasonable rooted tree topology, notably because it does not use any outgroup to root the tree. Such calculations in a Bayesian setting could simply be too heavy, thus supporting the use of simpler PAML-like approaches for estimating dates and rates from fixed rooted tree topologies.

Paradoxically, no such gap is observed for the date of the MRCA (Fig. 3b): the best distance-based methods, namely QPD* and LF*, find nearly the same point estimates and confidence intervals as BEAST used in a standard free-topology way, at least with a strict molecular clock (BSMC), that is, end of 2008. This date is compatible with Rambaut and Holmes (2009), Lemey et al. (2009) and Hedge et al. (2013) studies, but slightly older, as expected due to our larger data set incorporating more ancient strains. When using a fixed rooted tree topology, BEAST tMRCA becomes clearly older by 1 year or so, especially with a relaxed clock (BRMC*). However the discrepancy with distance methods involves only the MRCA and a few basal nodes (2 nodes with difference >6 months between QPD* and BSMC*, and 22 with BRMC*), while for most of the nodes the dates are highly similar (Pearson correlation coefficient with all node dates: QPD*/BSMC* ≈ 0.95, QPD*/BRMC* ≈ 0.91). The main difference among distance-based methods is between those using an outgroup to root the tree (LD*, QPD*, LF*, and RTT*) and the others (LD, QPD, and RTT) which infer the root position from the ingroup sequences only. The latter show more variability, larger confidence intervals, and tend to produce older date estimates, around the beginning of 2008 (these intervals and dates, however, are still statistically compatible with those of other methods). Again this larger variability is likely explained by the difficulty of tree rooting. Another factor for LD, and to some extent LD*, is the absence of temporal constraints: we see that their confidence intervals include a few root date estimates that are more recent (mid-2009) than our earliest strains (13 March 2009). This is clearly impossible and shows the advantage of incorporating temporal constraints, as in QPD and QPD*. With this data set, the solution of LD has ∼7% of branches such that the descendant node is older by 1 month or more than its parent (∼1.5% when the time difference is larger than 2 months, and ∼0.5% with 3 months).

To summarize, while the best distance-based methods (QPD* and LF*, used with FastME) are considerably faster than BEAST (especially QPD*, with negligible computing times), their dating results are quite similar. Regarding substitution rate estimation, we observe a large discrepancy between distance-based methods and BEAST, when used in the usual way estimating all parameters, including the tree topology and its root. However, with the fixed rooted tree topology, BEAST estimates of the substitution rate become similar to those of distance-based approaches.

## Discussion and Conclusion

We have described very fast algorithms to estimate rates and dates from serial data. These algorithms are based on a Gaussian noise, least-squares model, simplifying the Langley and Fitch's (1974) Poisson model implemented in the r8s package (Sanderson 2003). We showed that this model should be robust to uncorrelated violations of the molecular clock, and our simulation results confirm this theoretical prediction. LD uses a pure linear algebra approach, while QPD accounts for temporal precedence constraints, which appears to be important with real data. Given an input tree with dated tips, our algorithms provide the user with estimates of the substitution rate, the root date and the dates of all internal tree nodes, a task that is not achieved by RTT (also based on a simple, least-squares approach, but not able to date internal nodes). Our algorithms can be used to root the input tree when no outgroup is available, a feature that is not available in the r8s implementation of LF, and would be time consuming in the Poisson setting. Consequently, LD and QPD are also new fast, practical methods for tree rooting, which represent an alternative to the standard midpoint and minimum-variance approaches.

Computer simulations show that the accuracy of our algorithms is better than RTT's, and just slightly behind LF's with rooted trees. Compared to BEAST, our algorithms (combined with standard tree building methods) have a similar or better accuracy in estimating the substitution rate, while regarding dates the results depend very much on the presence of an outgroup and the way BEAST is used, estimating all parameters including the tree topology and its root, or using a fixed rooted tree topology. Globally, we did not observe any obvious limitation of our algorithms compared to BEAST, with simulated as well as real data sets. Moreover, our results clearly show the importance of having an accurate root position, a difficult goal when no outgroup is available and with relaxed (realistic) molecular clock.

Our algorithms require (quasi)linear computing times with rooted trees, as a function of n, the number of leaves. With unrooted trees, the computing time is (nearly) quadratic in n. This is obtained with complex algorithms, exploiting the closeness between least-squares and linear algebra; we also exploit the tree structure which makes it possible to design fast recursive procedures. This speed is important for current applications of phylogenetics. In Mourad et al. (2015), we analyzed a tree containing ∼24,000 dated HIV strains; running QPD* required ∼30 min on a standard desktop, while LF from r8s did not return any result after 2 weeks of computation. LSD has also been used by the members of the PANGEA_HIV consortium to study the phylodynamics of HIV epidemics in Africa using very large data sets (Fraser C., Ratmann O., personal communication; http://www.pangea-hiv.org/Projects/#phylodynamic, last accessed October 2015).

Our approach could be developed in several directions. First, we currently use a bootstrap approach to obtain confidence intervals, which is possible due to the speed of the algorithms, but still slow. Much faster approaches could be designed, for example, using the second derivative of the log-likelihood (least-squares) function. Second, we have described here the application of these algorithms to serial phylogenies with dated tips; easy adaptations should make it possible to use the very same approach to deal with phylogenies with time calibration points, attaching dated tips to ancestral nodes and using intervals (constraints) to account for ancestral date uncertainty. Last, an important direction is to implement fast methods that are able to cope with more complex, correlated molecular clock models, typically combining the least-squares framework with penalized criteria, similar to Sanderson (2002), or using some of our algorithmic solutions to deal with multi-normal approximations of the likelihood function (Thorne et al. 1998).

## Supplementary Material

Data available from the Dryad Digital Repository: http://dx.doi.org/10.5061/dryad.968t3, and from http://www.atgc-montpellier.fr/LSD/.
